# Pilot Scale Cavitational Reactors and Other Enabling Technologies to Design the Industrial Recovery of Polyphenols from Agro-Food By-Products, a Technical and Economical Overview

**DOI:** 10.3390/foods7090130

**Published:** 2018-08-21

**Authors:** Giancarlo Cravotto, Francesco Mariatti, Veronika Gunjevic, Massimo Secondo, Matteo Villa, Jacopo Parolin, Giuliano Cavaglià

**Affiliations:** 1 Dipartimento di Scienza e Tecnologia del Farmaco (DSTF), University of Turin, Via P. Giuria 9, 10125 Turin, Italy; francesco.mariatti@unito.it (F.M.); veronika.gunjevic@unito.it (V.G.); 2 ANDRITZ, Via C. Ravizza, 58, 20149 Milan, Italy; Massimo.Secondo@andritz.com; 3 Hydro Air Research Italia Srl, Strada Provinciale 181 n°11, 26833 Merlino, Italy; mvilla@hydair.com; 4 Tecnoimpianti Water Treatment Srl, Via S. D’acquisto 16/B, Pozzuolo M.na, 20060 Milan, Italy; jparolin@tecnoimp.com; 5 K & E Srl, Via S. Marco 1, 10023 Pessione, Italy; gcavagli@libero.it

**Keywords:** enabling technologies, pilot reactors, ultrasound, hydrodynamic cavitation, polyphenols, grape pomace, olive leaves

## Abstract

We herein provide an overview of the most recent multidisciplinary process advances that have occurred in the food industry as a result of changes in consumer lifestyle and expectations. The demand for fresher and more natural foods is driving the development of new technologies that may efficiently operate at room temperature. Moreover, the huge amount of material discarded by the agro-food production chain lays down a significant challenge for emerging technologies that can provide new opportunities by recovering valuable by-products and creating new applications. Aiming to design industrial processes, there is a need for pilot scale plants such as the ‘green technologies development platform’, which was established by the authors. The platform is made up of a series of multifunctional laboratories that are equipped with non-conventional pilot reactors, developed in direct collaboration with partner companies, in order to bridge the enormous gap between academia and industry via the large-scale exploitation of relevant research achievements. Selected key, enabling technologies for process intensification make this scale-up feasible. We make use of two selected examples, the grape and olive production chains, to show how cavitational reactors, which are based on high-intensity ultrasound and rotational hydrodynamic units, can assist food processing and the sustainable recovery of waste, to produce valuable nutraceuticals as well as colouring and food–beverage additives.

## 1. Introduction

Extraction is one of the essential food industry processes in the preparation of key ingredients. The choice of technology and procedure has a significant impact on food and beverage quality. A number of lab-scale and innovative protocols that make use of emerging technologies for food extraction and processing have been reported [1,2,3,4,5]. In spite of strong scientific evidence being produced, scale-up and further industrialization is generally troublesome, and industrial set-up requires a long period of study and optimization, even in the most promising of cases. The main reason for this is the large gap that exists between research laboratories and industrial plants. In the last two decades, few examples have been reported of pilot scale processes for the recovery of valuable by-products from industrial waste [6], as well as the use of emerging technologies for the isolation of food components [7,8] and essential oils [9]. It is worth mentioning that the Valréas eco-extraction platform (PEEV), a technical structure grouping equipment and expertise for R & D as well as small industrial series, runs in the field of plant value creation for the foodstuff industry and nutraceuticals [10]; an example of public and private partnership under the scientific guidance of Professor Chemat (Laboratoire Green de l’Université d’Avignon).

**Figure 1:**
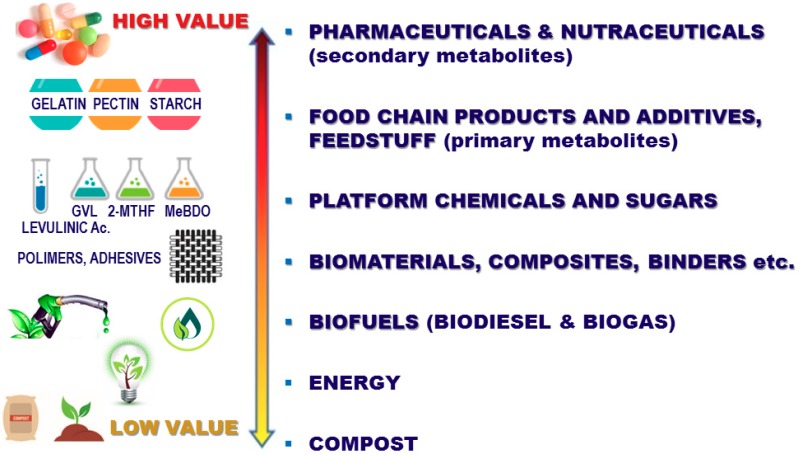
Creating a new value chain from agro-food waste.

In order to help bridge the gap between academy and large-scale production, the authors, a group of chemists, technologists, and process engineers, have worked together to set up the ‘green technologies development platform’, which brings together research, R and D, and production expertise. The reproduction of laboratory, gram-scale data in pilot, kilogram-scale reactors was a challenging project. This strategy enabled the primary heat and mass transfer data to be obtained, and a potential industrial plant to be designed. In this context, the growing need for the agro-food industry to selectively extract edible sources, smartly manage by-products, and adopt a biorefinery closing strategy has prompted the design of several cascade protocols and the creation of new value chains (Figure 1).

This approach fits well with the circular economy equation, and is a driving force for business opportunities. The development of green technologies for process intensification is a prerequisite for advancing the biorefinery concept and green extraction [11].

## 2. The State of the Art in Agro-Food Extraction 

**Figure 2:**
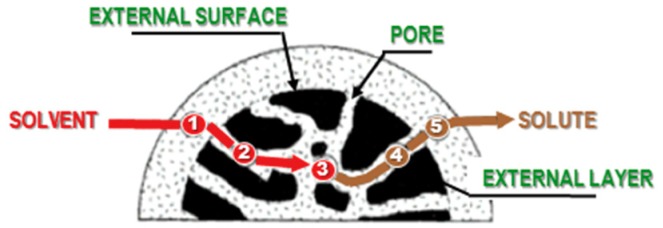
Liquid-solid extraction diagram.

The main technique used to extract flavourings, colourings, and bioactive compounds from natural matrices is liquid–solid extraction, during which a solute is removed from a solid matrix using a solvent or a supercritical fluid, according to essentially diffusion- and osmosis-based principles [12,13]. In general, liquid–solid extraction involves the following fundamental phenomenological steps: (i) solvent diffusion from the solution bulk to the solid surface; (ii) solvent penetration into the micro-/macro-porosity of the solid; (iii) dissolution, into the solvent, of the solute present in the solid; (iv) diffusion of the solute towards the surface of the solid, which is caused by differences in the solute concentration in solution, as it is more concentrated inside the matrix than outside the solid; and (v) diffusion of the solute through the boundary layer into the bulk solution (Figure 2). The extraction process, and therefore the diffusion, stops when a condition of equilibrium is established between the solute concentration within the solid and the solute concentration outside of it.

The diffusion speed of the solute molecules up to equilibrium, from higher concentration to lower concentration zones, is governed by Fick’s law. Long extraction times and prolonged heating increase the risk of food component degradation, which causes a partial loss in the functional and flavouring properties [2,14,15]. In general, the process is completed with the mechanical separation of the solution, which is rich in the extracted compounds, from the exhausted solid matrix.

Static maceration, which is maceration with agitation and percolation, is among the most traditional techniques for the extraction of bioactive compounds from vegetal matrices [16,17]. In this technique, the solid matrix to be extracted is put into contact with a liquid solvent phase, which, in most cases, consists of a hydroalcoholic solution with ethanol contents that may vary from 40% to 80% [18].

It should be noted that the liquid–solid extraction benefits from efficient and close matrix–solvent contact, and techniques have been developed to optimize this aspect. However, traditional techniques have limits that cannot be overcome, namely the need for organic solvents; mass transfer coefficients beyond certain levels cannot be obtained, meaning that it is impossible to reduce extraction phase times below 1–2 h, and that the hold-up of solvents in extraction devices consequently becomes non-negligible [19]. This is the minimum time for conventional extractions, including percolation, continuous stirred tank reactors (CSTR), and continuous screw type countercurrent systems. For intensified processes with cavitational reactors, the required extraction time is <15 min, owing to the maximization of the mass transfer coefficient generated by cavitation, and of course, the magnification of the interphase area, which is again a consequence of cavitation.

The mass transfer general equation is Fp = A × K × ΔC (Fp—mass flus; A—interphase area; K—mass transfer coefficient; ΔC—driving force).

Cavitational extraction systems and process intensified systems enhance both A and K, as documented by the literature [20,21].

## 3. Improved Extractions under Ultrasound and Hydrodynamic Cavitation

**Figure 3:**
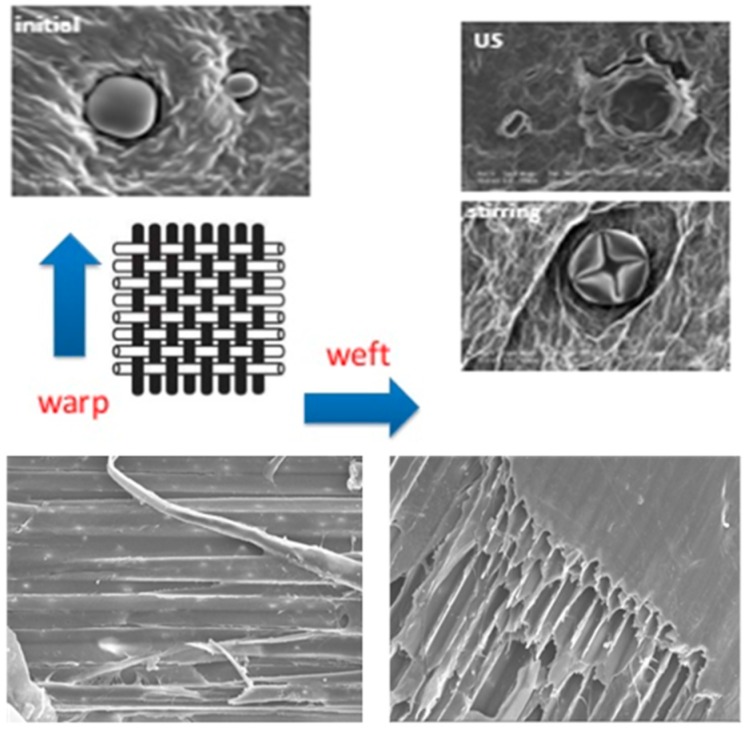
Dismantling biomass structure under hydrodynamic cavitation (HC) and ultrasound (US), like a fabric where ‘warp’ and ‘weft’ (from the authors’ laboratory).

Low frequency ultrasound (US) and hydrodynamic cavitation (HC) are key enabling technologies for process intensification, and are unique non-thermal techniques that see widespread use in food extraction and processing [22,23,24,25]. The growing interest in food sonoprocessing over the last two decades has been well documented in a number of reviews and books [23,26,27]. By contrast, low frequency US (20–200 kHz) employs power levels that are high enough to generate cavitation and is therefore capable of producing physical and chemical modifications in a variety of applications. The food industry is always looking for innovative technologies that can enhance processing efficiency, reduce energy consumption, and produce high-quality, safe products, which is why US technology feasibility has been widely investigated. In recent years, several examples of US being used to process and interact with liquid foods, primarily with dairy and fruit juices, have been reported in the literature. Mechanical and shear forces, agitation, microjets, microstreaming, cavitation hot spots, and shockwaves are some of the physical forces that have been effectively used in several food processing applications [22]. Cavitation phenomena (acoustic and/or hydrodynamic) are of great importance in food extraction and processing, as they are fast and efficient. As depicted in Figure 3, both of the glands that are on the surface and those deeper within the solid vegetal structures are easily opened and dismantled, leading to a much faster cytoplasmic component recovery [28].

**Figure 4:**
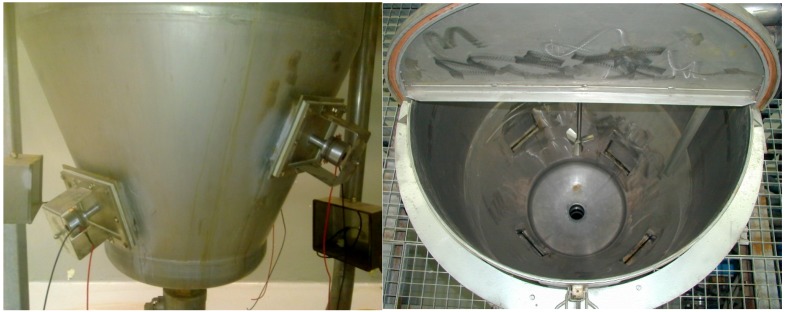
External and internal view of an industrial batch US reactor (300 L) (with kind permission from Martin Bauer, Italy).

**Figure 5:**
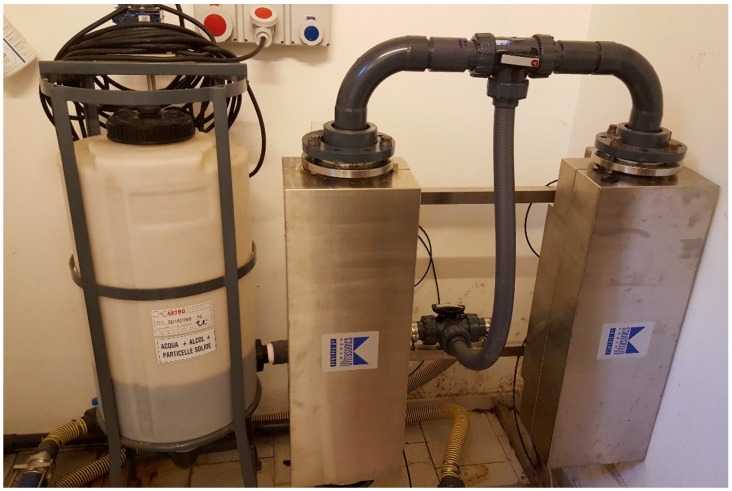
Double-unit, high-power sonochemical reactor (25 kHz and multi-frequency 80–120 kHz) (‘green technologies development platform’, Dipartimento di Scienza e Tecnologia del Farmaco (DSTF), University of Turin, Turin, Italy).

While US protocols are safe and can operate at low temperatures, profitable applications require dedicated batch-mode (Figure 4) or flow-mode (Figure 5), high-throughput equipment. Flow-mode designs lend themselves well to large biomass volumes, as do rotor/stator HC generators [29], in which residence time becomes the main parameter by which the treatment types are discerned. While high-intensity US systems become ever more standardized, the means by which energy is applied to the medium (flow cell design and number of transducers) is unique to every application [30,31], thus offering opportunities for patent protection, and enticing fresh investment.

Theoretical studies and experimental evaluations carried out by several authors have underlined that the most promising and scalable innovative process intensification technique for the extraction of valuable food components is the controlled cavitation-assisted extraction [32,33]. The term ‘cavitation’ refers to the liquid–steam transition phase, which occurs under isothermal conditions, caused by reducing the pressure to the steam pressure value in the liquid phase. Controlled cavitation can be generated by both acoustic and hydrodynamic means. In US-generated cavitation (acoustic cavitation or sonication), acoustic waves, generated by sonotrodes, propagate within the fluid medium (extracting solvent), causing air bubbles and mechanical movements of low amplitude (in the order of microns) and high frequency (in the order of tens of thousands of oscillations per second) to be generated.

The propagation of an acoustic wave, especially a US-generated wave, within a fluid leads to sinusoidal pressure oscillation. The first part of the wave is a voltage wave, which generates a series of bubbles (in the case of water and aqueous solutions, generally with a radius of between 10 and 200 microns), while the second part, which follows immediately afterwards, is a compression wave and causes violent bubble collapse. Bubbles therefore form, grow in size, and collapse over just a few microseconds. The high pressures and temperatures generated when the bubbles collapse in turn generate micro-jets that are oriented towards the solid surface. These jets thus break the cell walls, meaning that the cell content is released into the extraction medium [32]. Cavitation is favoured at low temperatures and when the chemical–physical conditions of the cavitational-system hold-up are to induce cavitation and the consequent generation of bubble swarms in a liquid mass. For an external observer, it is as if the system were at a temperature (T) and a pressure (P) that are significantly higher than the average of the system itself [34]. This happens precisely because the portion of chemical transformations that takes place within each micro bubble, during its life-span, does so at T and P conditions that are a few orders of magnitude higher than the system average.

The justification for the increase in the extraction performance seen in US and HC is to be found in mass transfer intensification and in the facilitation of solvent access to plant cells. Sound waves with frequencies above 20 kHz lead to significant increases in extraction performance, as they cause alternate expansion and compression in matrices, the formation of micro-bubbles, and thus intensify solvation into matrix cells. In the case of HC, controlled cavitation is generated by cavitational elements, known as hydrodynamic cavitators, for example, of the ‘rotor–stator’ type, in which the rotor element generates enough speed within the liquid to generate cavitation. Controlling cavitation means controlling the dynamics of the generated vapour bubbles, making them dynamically implode in a limited area and at a higher pressure. It is thanks to the high local temperatures and pressures that are dynamically generated during the implosion phase of the bubbles that controlled cavitation can be efficiently used to extract high added-value compounds from solid vegetable matrices. A controlled HC device can impose high edgeways stress on a liquid that passes through it. This stress produces micro-bubbles in the liquid, causing asymmetric bubble implosions that produce high local temperatures and pressures that can be maximized using a multi-rotor configuration, in which several disks of proper geometry, connected to a motor shaft, rotate inside a confined chamber and generate friction with the liquid, thus creating cavitation [35]. The solid matrix containing the compounds to be extracted is then subjected to the action of various phenomena. Pressure and temperature shocks, edgewise stress, and turbulence disrupt it and make the compounds that are to be extracted from it more accessible. Therefore, feeding a solid–liquid phase matrix dispersion into a dynamic type quarry operator leads to certain matrix disintegration, the generation of an interface area and an increase in matrix porosity volume. As a consequence, matrix micromixing with the liquid phase ensures the optimisation of the extraction yield in a continuous process. Moreover, a multi-rotor HC extractor allows for the liquid and solid phases to be fed into the system countercurrently in a multistage operation, in which three physical rotor–stator stages correspond to >15 theoretical balance stages. This allows the matrices to be perfectly depleted of the relevant substances, and for the liquid phase flow rate to be kept to a minimum [35]. Ultrafast processes avoid the degradation risk of active principles because of the release of strongly oxidizing radicals [36].

## 4. Flow-Mode Extraction in Pilot Scale

As a result of the simpler continuous operation, a flow-mode extraction process would be very welcome by the industry. Nevertheless, the lack of suitable equipment, data, and experience has always hindered any development. In the last few years, we introduced one of the first example of pilot scale US-assisted extraction using a double-unit US device developed by WEBER Ultrasonics AG (Karlsbad, Germany). A number of flow devices, which have been previously described and tested, were also used [37,38]. The rotor/stator HC units used were the Turbex^®^ (BOB Service Srl, Turin, Italy) multistage counter-current quarry plough [35] and the system Rotocav^®^ (EPIC, Mongrando, Italy) [39,40]. In previous works, we compared rotor/stator HC generators with classic orifice plates and Venturi tubes [41], which have several applications in food processing [33,40,41]. However, with regards to the treatment of biomass and plants extraction with irregular particles in suspension, the risk of orifices and tubing clogging discourage the application at least with orifice plates, preferring rotor/stator HC generators [42].

**Figure 6:**
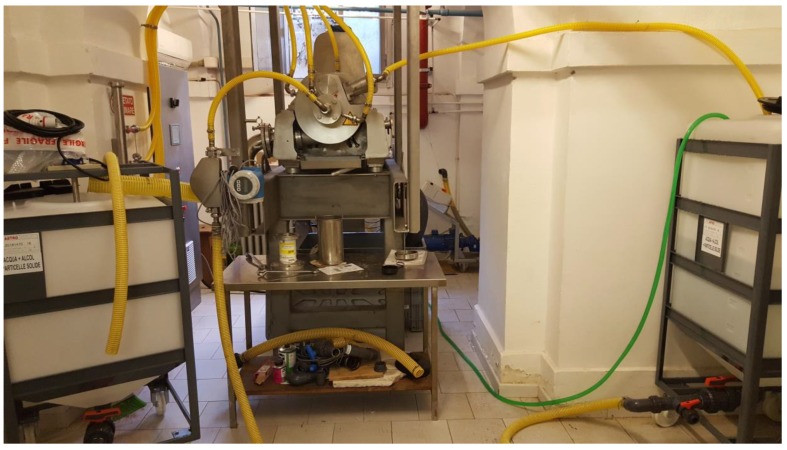
Decanter F2000 ANDRITZ (‘green technologies development platform’, DSTF, University of Turin, Turin, Italy).

**Figure 7:**
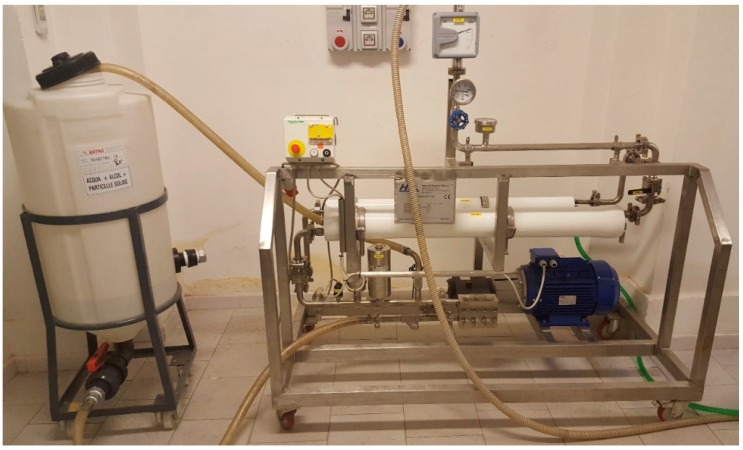
Hydro Air Research Italia pilot unit for ultra-, micro-, and nano-filtration (‘green technologies development platform’, DSTF, University of Turin, Turin, Italy).

**Figure 8:**
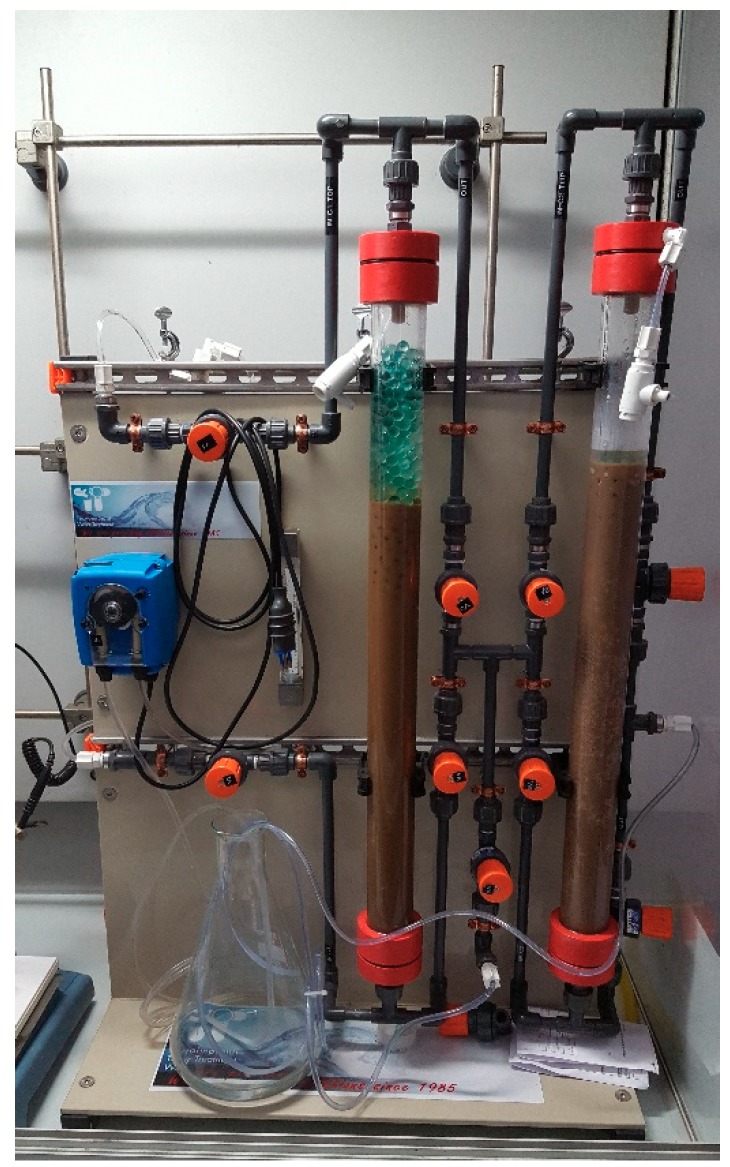
Skid Tecnoimpianti Srl for resin separation (‘green technologies development platform’, DSTF, University of Turin, Turin, Italy).

The suspension recovered from the cavitational extractors was subjected to separation in decanters and centrifuges that were supplied by ANDRITZ (Milan, Italy) (Figure 6) [43], concentration over membrane systems that were supplied by Hydro Air Research (Merlino, Italy) (Figure 7) [44], and in suitable resin columns supplied by Tecnoimpianti (Milan, Italy) (Figure 8) [45].

The final evaporation was either performed on a 50 L rotary evaporator (Keda, Shanghai, China), or on larger-scale vacuum drum dryers supplied by ANDRITZ (Milan, Italy) [43]. Two different sub-streams of food waste, grape pomace and olive leaves, have been extracted using efficient cavitational reactors and have been evaluated in both lab- and pilot-scale experiments for potential full-scale implementation.

## 5. Main Features of a Pilot Scale Green Technologies Development Platform

The green technologies development platform (University of Turin, Turin, Italy) consists of a series of multifunctional laboratories and was established in direct collaboration with ANDRITZ (Milan, Italy), Hydro Air Research Italia, Tecnoimpianti, and K&E. The platform has been designed to bridge the gap between academic research knowledge and industrial R and D development, with the aim of efficient technology transfer and the industrialization of innovative processes.

Main results currently achieved by the platform are listed below, as follows:

(i) The efficient and selective extraction of the primary and secondary metabolites (e.g., starches, cellulose, pectins, polyphenols, anthocyans, flavonoids, etc.) from agro-food matrices and production waste, in particular from grape pomace and olive leaves.
(ii) Multi-stage processing in the food, nutraceutical, pharmaceutical, and flavouring fields, including a transition from lab-scale data to pilot-scale preparation, and the subsequent design of potential large-scale production plants.

Investigations of the ‘lab-scale to pilot reactors’ transition shows that continuous and semi-continuous cavitational-assisted extraction processes lead to exceptional process intensification that is very much in line with the principles of green extraction and the circular economy equation. Both grape pomace and olive leaves have been efficiently extracted exclusively in water alone, in the absence of ethanol, and in the presence of other organic solvents. The efficient and fast separation, purification, concentration, and drying furnished high quality green extracts that are rich in polyphenols and provided useful data for industrialization.

**Figure 9:**
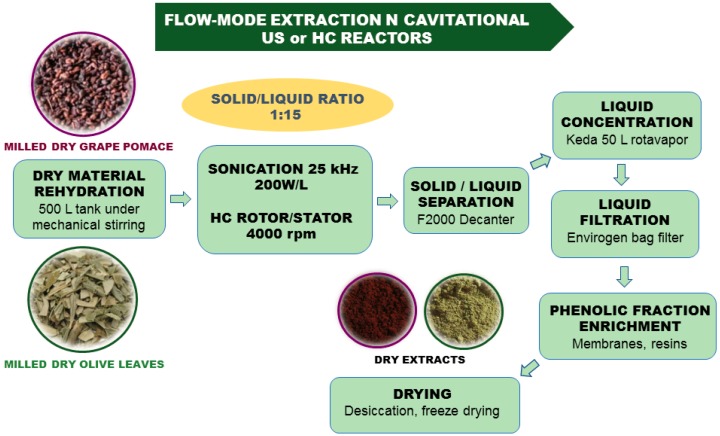
Pilot scale extraction processes flow chart.

The standard procedure in pilot scale is depicted in Figure 9. The dry material (particle size 0.2–0.4 mm) is rehydrated for 1 h in pure water, then, the suspension under mechanical stirring (solid:liquid ratio of 1:15) is pumped in either the US reactor or the rotor:stator HC unit. The treated suspension is subsequently pumped in the F2000 decanter ANDRITZ (Milan, Italy), g-force of 3000 to 3500 g), which can process up to 2 m^3^/h. The liquid fraction is concentrated to half volume under a vacuum in a 50 L rotavapor, and is filtered using a bag filter device (Envirogen). The clear solution is subjected to membrane micro-filtration to eliminate inorganic salts and concentrate the polyphenols [46,47]. Analogously phenolics can be adsorbed on suitable resins and recovered with ethanol [48].

Several comparisons between batch the US-assisted extraction (25 L and 250 L reactors) and flow US-assisted process showed the better performance of the latter, both in terms of time processing and extraction yield, namely up to 50% of time saving and 4–25% higher yields (unpublished data, patent pending).

Starting from 1 t of cellar pomace (at 45% humidity), it is expected that 20 kg of dry extract, which is rich in polyphenols and valued at 40 €/kg if sold, wholesale, in drums, will be obtained.

Starting from 1 t of olive leaves, it is expected that 80 kg of dry extract, which is rich in oleuropein and more polyphenols, and is valued at 30 €/kg if sold, wholesale, in drums, will be obtained. For example, the average energy consumption (extraction step in pure water) needed to obtain the dry extract from the olive leaves with a content of 21.5% weight of oleuropein (HPLC), was about 0.4 kWh/kg of the dry extract.

## 6. Features of Grape Pomace and Olive Leaves Polyphenols

### 6.1. Polyphenols

Polyphenols are a large family of secondary metabolites that are produced by plants as a defence against external factors, such as UV radiation, pollution, and parasite attacks. Complex mixtures of metabolites form phytocomplexes that maximize the protective effect of individual molecules through synergetic and complementary activities [49,50]. This pool of natural molecules, in particular polyphenols that are either extracted from plants or from agro-food industry waste processing, have recently aroused a great deal of market interest as antioxidant products that can be used in various sectors as additives, especially in the food industry. The most important groups of polyphenols that affect the sensory appearance and properties of food are anthocyanins, which are responsible for the red/purple colour of fruits and flowers, and catechins and proanthocyanins, which, like their tannin precursors, are responsible for colour, astringent taste, and aromatic notes. Flavonoids, on the other hand, are known for their blood vessel and capillary microcirculation protection function. There is no evidence to suggest that plant-derived polyphenols have any toxic effect on the human diet, although their role in human metabolism is still under study.

### 6.2. Grape Polyphenols

Polyphenolic substances are mostly localized in the solid structural fractions that are present in grapes, and therefore in the pomace. Grape polyphenols can be divided into two classes, flavone and gallic acid derivatives, which are generally found as glucosides [51]. The phenolic compounds of major nutraceutical interest are (a) resveratrol, (b) anthocyanins, (c) condensed tannins and catechins, and (d) quercetins. These are all compounds that show a protective action against the oxidation of low-density lipoproteins (LDL) in blood circulation. Anthocyanins are flavonoids that are concentrated in grape skin. They are strong radical scavengers with chemopreventive properties. About 50–70 mg per 100 g of red grapes is the amount of anthocyanins that can be obtained. Condensed tannins, also known as proanthocyanidins, are present in the skin and, in higher amounts, in the seeds. The recommended dose for beneficial effects is 1–3 mg of proanthocyanidins per kg of body weight.

### 6.3. Olive Leaf Polyphenols 

Olive leaves are a rich source of secondary metabolites, such as secoiridoids and phenols. Secoiridoids are chemical compounds that contribute to the lowering of blood pressure and blood glucose levels in diabetic individuals. Oleuropein is one of the most abundant metabolites that can be isolated from olive leaves, and it has the characteristic of improving the metabolism of lipids, thus reducing the causes of obesity. It is also a chemopreventive. Olive leaves have recently been included in the European Pharmacopoeia (80% ethanol extract). Secoiridoids are the main compounds in olive leaves, and their particular chemical structure derives from that of iridoids. Oleuropein shows poor bioavailability, is non-toxic, and has no side effects [52].

### 6.4. Flavonoids

Flavonoids are almost ubiquitous in the plant kingdom. Flavones (luteolin-7-glucoside, apigenin-7-glucoside), flavonols (rutin, quercetin, catechin), and flavanonols (taxifolin) have been identified in the leaves of *Olea europaea*. As has been observed in secoiridoids, flavonoids also have high antioxidant, anti-inflammatory, antimicrobial, and antiviral activity. The antiradical action exerted by oleuropein, hydroxytyrosol, tyrosol, rutin, and elenolic acid is significant and relevant [53].

### 6.5. The Polyphenol Market

As stated in the ‘Polyphenols Market Size and Share—Industry Report 2024’ [54], the worldwide demand for polyphenols in 2015 was 16,400 tonnes, which corresponds to total turnover of 760 million USD. Furthermore, it has been estimated by various expert analysts in the field, that, between 2016 and 2024, an increase in the demand for polyphenols equalling 8.5%/year in terms of volume and 6.3%/year in terms of turnover will be observed. This will culminate in an absolute value of 34,000 tonnes, which corresponds to 1200 million USD in terms of turnover, in 2024. The market share in 2015 was dominated by polyphenol-rich extracts from grape pips, with a market share of 52% (in terms of turnover); followed by polyphenol-rich extracts from green tea, with a share of 24%; and then by apple-rich extracts, with a share of 12%. The remaining 12% was found in polyphenol-rich extracts from other matrices (i.e., cocoa, coffee, hazelnuts, tomatoes, olive leaves, red fruits, rosemary, carob, etc.). Green tea, with an expected annual growth of 9.6%/year from 2016 to 2024, is the market sub-segment where the highest growth rate is expected.

The significant health effects of introducing polyphenols into the daily diet, both with food and in the form of food supplements, have been widely demonstrated. Countless scientific studies have shown that polyphenols have anti-aging, anti-inflammatory, antihypertensive, cholesterol-lowering, and chemopreventive properties. Furthermore, they are still widely used in cosmetic preparations, even for topical use, because of their anti-ageing properties.

In addition to the above, the progressive increase in the average age of the population of rich or high gross domestic product (GDP) countries is a real driving force for polyphenol demand. It is on this factor that the progressive increase in the importance of chronic diseases, such as cardiovascular diseases, neurodegenerative diseases (i.e., Alzheimer’s, Parkinson’s, etc.), and osteoporosis, depends. In addition, the increased focus on the physical condition of the geriatric population also contributes to the growth of the global demand for polyphenols. The demand for food and nutraceuticals that are reinforced with vitamins and probiotics as key ingredients for prevention, has also significantly increased over recent years. Consumers in rich countries are increasingly inclined to spend money on healthy nutrition and food supplements as a source of general well-being, all of which turns into an improvement in the market share of polyphenol-rich products.

Polyphenols are also important as ingredients in functional foods and beverages. Functional drinks, in particular, are the category with the highest market share and the highest growth rate.

**Figure 10:**
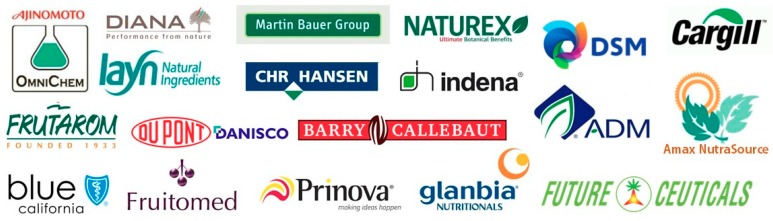
The leading companies in the world market for polyphenols.

The Asia-Pacific region was the region with the highest polyphenol consumption in 2015, with a 40% share. The increase in the geriatric population in Japan and China is expected to be the main factor upon which the main portion of growth in the market demand for polyphenols will depend over the next decade. In Figure 10, the company logo of the main industrial suppliers of polyphenols are depicted.

## 7. Conclusions

The design of an efficient industrial recovery of polyphenols from agro-food by-products requires data from pilot plant experiments. Besides the eco-extraction platform PEEV in France, we described herein the ‘green technologies development platform’ in Italy, which can be a source of innovation, thanks to the complementary expertise of its partners and the knowledge generated by its joint research and development activity. The flow-mode extraction processes using cavitational reactors, enable an easier scaling up to industrial production. The examples shown here illustrate how it is possible to tackle studies up to a technology readiness level (TRL) of six–seven, with the development of a pilot plan and scaling up, and thus reducing the gap between the research labs and the industry within an innovative technological process that sees industrial business as a driving force.
